# Antioxidant Properties of Alpha-Lipoic (Thioctic) Acid Treatment on Renal and Heart Parenchyma in a Rat Model of Hypertension

**DOI:** 10.3390/antiox10071006

**Published:** 2021-06-23

**Authors:** Ilenia Martinelli, Daniele Tomassoni, Proshanta Roy, Lorenzo Di Cesare Mannelli, Francesco Amenta, Seyed Khosrow Tayebati

**Affiliations:** 1 School of Pharmacy, University of Camerino, 62032 Camerino, Italy; ilenia.martinelli@unicam.it (I.M.); francesco.amenta@unicam.it (F.A.); 2 School of Biosciences and Veterinary Medicine, University of Camerino, 62032 Camerino, Italy; daniele.tomassoni@unicam.it (D.T.); proshanta.roy@unicam.it (P.R.); 3 Department of Neuroscience, Pharmaceutical and Child Health Area (NEUROFARBA), University of Florence, 50139 Florence, Italy; lorenzo.mannelli@unifi.it

**Keywords:** alpha-lipoic acid, hypertension, kidney, heart, parenchyma

## Abstract

Renal and cardiac impairments are frequent events in the presence of hypertension. Organ damage is mainly linked to oxidative stress due to high blood pressure and may be reduced by antioxidant supplementation. Alpha-lipoic acid (ALA) is one of most effective antioxidants. It is widely used as a nutritional supplement in a racemic mixture (+/–), even though the (+)-enantiomer is biologically active. This study was designed to investigate the effect of treatment with (+/–)-ALA and its enantiomers on renal and heart parenchyma in spontaneously hypertensive rats (SHR), using immunochemical and immunohistochemical techniques. The results confirmed that the oxidative mechanisms of organ alterations, due to hypertension, and characterized by glomerular and tubular lesions, left ventricular hypertrophy, and fibrosis but not by apoptosis were accompanied by proteins’ and nucleic acids’ oxidation. We found greater effectiveness of (+)-ALA compared to (+/−)-ALA in reducing oxidative stress, cardiac and renal damages in SHR. To conclude, these data propose (+)-ALA as one of the more appropriate antioxidant molecules to prevent renal and cardiac alterations associated with hypertension.

## 1. Introduction

Hypertension is a multifactorial disease characterized by elevated blood pressure. It is a risk factor for renal and cardiovascular diseases [1,2]. Both the kidney and the heart are target organs in hypertension, and their structure and function become gradually impaired with longstanding hypertension. Predictably, as a consequence of hypertension, the concomitant presence of renal and heart failure in the same patient was reported [3]. Indeed, cardiorenal interactions occur through several pathways in both directions [4].

Multiple mechanisms are implicated in the determination of hypertension-induced kidney damage. These involve different cell types and anatomical structures in the kidney, including the glomeruli, tubulointerstitium, immune cells, and vasculature. Arterial hypertension is correlated with a progressive intimal thickening of muscular arteries and arterioles of the renal parenchyma, due to collagen deposition and spreading of elastic fibers and myofibroblasts [5]. Other histopathological alterations are interstitial fibrosis, associated with tubular atrophy, and arteriosclerosis of the afferent arterioles [1,5].

Concerning the mechanisms, the hypertrophy of the cardiac muscle is one of the most important maladaptive responses to augmented workload. It is associated with histological and ultrastructural modifications and alterations in the level of different enzymes [6]. In addition to cardiomyocyte hypertrophy, myocardial microcirculation rarefaction, interstitial fibrosis, and myocyte apoptosis are reported [7].

Increasing relevance is attributed to the role of oxidative stress for what concerns the mechanisms of hypertension and cardiovascular and renal damage because it promotes endothelial dysfunction, vascular remodeling, and inflammation, leading to vascular damage [8,9]. Excess bioavailability of reactive oxygen species (ROS) often accompanied hypertension-induced structural abnormalities on mitochondria of the cardiomyocyte and renal parenchymal cells [10]. In experimental and human hypertension, an increase in the production of superoxide anions and hydrogen peroxide, a decrease in nitric oxide (NO) synthesis, and a reduction in endogenous antioxidant bioavailability have been observed [11,12]. Other biomarkers of oxidative stress in human hypertension are isoprostanes and malondialdehyde (MDA) [13].

The most effective treatment in the management of hypertension seems to be the administration of anti-hypertensive drugs with antioxidant properties [14]. Even if the data of clinical trials are discordant, the supplementation of natural antioxidants could also be a promising therapeutic tool for hypertension [15,16]. Experimental evidence suggests that early antioxidant treatment may induce beneficial effects on hypertension and cardiovascular and renal damage [17,18]. However, antioxidants are less effective in the advanced stage of hypertension, whereas the renin–angiotensin system (RAS) may still play a central pathogenetic role, even at later stages, as confirmed by the positive action of its blockade [19].

Previously, we demonstrated that the antioxidant alpha-lipoic acid (ALA) treatment reduced oxidative stress and prevented adhesion molecule expression in the cardiac and renal vascular endothelium of spontaneously hypertensive rats (SHR) [20]. The SHR is a reliable model of a spontaneously developing pressure load similar to human essential hypertension; thus, it is usually recognized that the SHR represents an analog of essential hypertension in humans [21,22]. Severe hypertension arises in 100% of SHR with a systolic blood pressure of 180 mmHg or higher [23].

In SHR, we already assessed the neuroprotective activity of ALA in central nervous system lesions consequent to peripheral nerve injury [24]. Recently, data from the spinal cord could explain the protection of ALA on the brain [25]. Finally, the use of ALA for therapeutic purposes was reviewed by [26].

Therefore, the current study aims to investigate the alterations in renal and heart parenchyma of SHR and the potential benefits of ALA on it, using immunochemical and immunohistochemical techniques.

## 2. Materials and Methods

### 2.1. Animals and Tissue Treatment

SHR has a genetic predisposition to develop arterial hypertension, allowing it to study causes, mechanisms, pathology, and behavioral consequences of the disease [27]. Twenty-week-old male SHR (*n* = 30) and age-matched normotensive Wistar–Kyoto rats (WKY) rats (*n* = 6) were used and treated, as previously described [20]. Animal manipulation was carried out according to the National and European Community Guidelines for Animal Care (DL 116/92, of application of the European Communities Council Directive, 86/609/EEC) and also following the ethical guidelines of the University of Florence. The guidelines deal with the treatment of animals under lab testing. These guidelines are consistent with the Guide for the Care and Use of Laboratory Animals of the US National Institutes of Health (NIH Publication No. 85-23, revised 1996; University of Florence assurance number: A5278-01). Experiments involving animals have been reported according to ARRIVE guidelines. All efforts were made to minimize animal suffering and to reduce the number of animals used.

Concerning the treatment, alpha-lipoic acid (1,2-dithiolane-3-pentanoic acid or thioctic acid), exists in two enantiomers, namely (+)- and (–)-alpha-lipoic acid. It is due to the presence of an asymmetric carbon in the C3 position. The former enantiomer represents the active form of the compound [20].

Lipoic acid, as (+/−)-compound, lysine salt (+)-enantiomer and (−)-enantiomer, was provided from Sintactica (Milan, Italy). Compounds were solubilized in NaOH-supplemented physiologic solution and buffered to 7.4 pH by adding HCl. Different racemic and lipoic acid salt compounds were solubilized in saline [25] and SHR rats were treated once a day for 30 days with an i.p. injection of 250 µmol/kg/day of (+/–)-ALA (*n* = 6); 125 µmol/kg/day of (+/–)-ALA (*n* = 6); 125 µmol/kg/day of (+)-ALA (*n* = 6) and 125 µmol/kg/day of (–)-ALA (*n* = 6). WKY (*n* = 6) and SHR (*n* = 6) rats were also treated with the same amounts of the vehicle as control. Food intake and body weight were monitored daily, while measurements of systolic blood pressure were performed once a week, in conscious rats, by tail-cuff methods using electronic sphygmomanometer, specific for small animals (Model: GIMA Italy, B3Plus). Animals were anesthetized and then perfused [20]. After, the kidney and heart were dissected out, weighed, fixed for 72 h in 4% paraformaldehyde, dehydrated with alcohol and embedded in paraffin wax. Longitudinal consecutive renal and heart sections were cut using a rotary microtome and processed for morphological analysis and immunohistochemistry (IHC), as detailed previously [20].

### 2.2. Histological Analysis, Immunohistochemistry and Immunofluorescence

Paraffin blocks were cut into 6–8 μm sections for Masson staining and immunohistochemistry. Kidney tissues were also stained with periodic acid-Schiff (PAS) to highlight basement membranes of glomerular capillary loops and tubular epithelium, and semiquantitative analysis of the glomerular intensity of PAS staining was performed. Moreover, glomerular and tubular injury scores were examined in Masson’s stained sections as follows. For the glomerular injury score (GIS), approximately 60 random glomeruli from each specimen were examined and each glomerulus was graded from 1 to 4 [28]. Then, each score was calculated according to the formula GIS = [(1 × number of grade 2 glomeruli) + (2 × number of grade 3 glomeruli) + (3 × number of grade 4 glomeruli)] × 100/(number of glomeruli observed) [29,30]. The tubular injury was defined using the scoring system from 0 to 4 [31]. Then, the tubular injury score (TIS) was calculated according to the formula TIS = [(1 × number of grade 1 dark tubules profiles area) + (2 × number of grade 2 dark tubules profiles area) + (3 × number of grade 3 dark tubules profiles area) + (4 × number of grade 4 dark tubules profiles area)]/(surface area examined) [30]. Cardiac fibrosis analysis was performed by measuring the presence of accumulation of connective tissue in the parenchyma of the left ventricle and in the adventitia of the coronary arteries in Masson trichrome-stained sections from each heart [32].

Immunohistochemistry anti-8-hydroxy-2′-deoxyguanosine (anti-8-oxo-dG) was performed both in the kidney and heart using a mouse monoclonal antibody (Trevigen, Gaithersburg, MD, USA, Cat. No. 4354-MC-050) diluted 1:250. This mouse monoclonal antibody specifically binds to 8-oxo-dG within DNA, as detailed [25]. The product of the immune reaction was then revealed by exposing slides with anti-mouse biotinylated polyclonal antibody (Merck-Millipore, Burlington, MA, USA, Cat. No. AP124B) (diluted 1:200). The colored reaction product was developed with 3′,3′-diaminobenzidine tetrahydrochloride (DAB) solution (Vector Laboratories, Inc., Burlingame, CA, USA). The sections were observed, and images were captured with the microscope (Leica) and evaluated using an IAS 2000 (Delta Sistemi, Rome, Italy). The number of 8-oxo-dG positive cells was counted per high-power field (HPF) images (40× magnification).

Finally, the Membstain Apoptosis kit Direct (MBL International Corporation, Woburn, MA, USA, Cat. No. 8445) based on in situ labeling of nuclear DNA fragmentation (Tolt-mediated dUTP nick-end labeling, TUNEL staining) was used following the company instruction. The sections were analyzed under a microscope a fluorescence (Olympus Italia, Segrate, Italy), using a 465 nm excitation filter. The number of TUNEL positive cells was counted per HPF images (20× and 40× magnification for heart and kidney, respectively).

### 2.3. Protein Extraction and Western Blot Analysis

Protein lysate was obtained homogenizing the tissue using lysis buffer as previously detailed [32]. For 8% and 12% SDS-PAGE, 40 µg total protein sample was deposited in each well. The separated protein was transferred to a nitrocellulose membrane, which was blocked with bovine serum albumin (BSA) in PBS 0.1% Tween-20 for 1 h at room temperature and then incubated with anti-caspase-3 (Cell Signaling Technology, Danvers, MA, USA, 1:1000) and a GAPDH antibody as an internal reference (Cell Signaling Technology, Danvers, MA, USA, 1:1000) at 4 °C overnight. Then, the membranes were transferred to room temperature and incubated with horseradish peroxidase-conjugated goat anti-rabbit or goat anti-mouse secondary antibodies (Bethyl Laboratories, Inc., Montgomery, TX, USA, dilution 1:5000).

Moreover, the protein oxidation status was investigated using the OxyBlot Protein Oxidation Detection kit (Merck-Millipore, Burlington, MA, USA, Cat. No. S7150). The detection was performed using the LiteAblot PLUS kit (EuroClone, Milan, Italy). Band intensities were measured by densitometry with IAS 2000 image analyzer (Biosystem, Rome, Italy). Blots are representative of one of three separate experiments.

### 2.4. Statistical Analysis

All data were expressed as mean ± S.E.M. The statistical comparisons were determined using Student’s t-tests for WKY and SHR control (C) and one-way ANOVA for SHR C and groups of SHR with ALA, followed by the Newman–Keuls post hoc tests for multi-groups, with *p*-value < 0.05 being considered statistically significant.

## 3. Results

No significant differences were observed between WKY and SHR or SHR treated with ALA in body weight. Neither hypertension nor pharmacologic treatments modified kidney weight values. The ratio of heart weight to body weight was increased in control SHR rats (3.37 ± 0.12) compared to control WKY rats (3.05 ± 0.06). The antioxidant treatment did not change this phenomenon. At sacrifice, the systolic blood pressure values were higher in control SHR rats (215 ± 3 mmHg) compared to the normotensive WKY rats (148 ± 9 mmHg). In SHR rats, 30 days of treatment with any stereoisomer of ALA did not significantly affect systolic blood pressure levels [20].

### 3.1. Kidney

**Figure 1:**
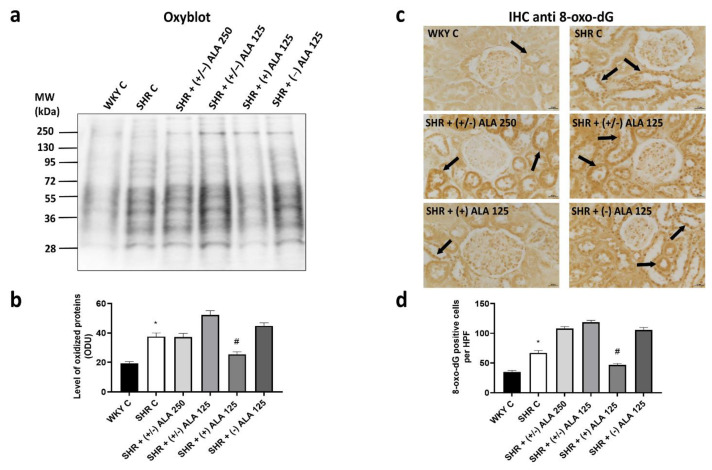
Oxidative stress in the kidney. (**a**) Lysates of kidney from WKY C, SHR C, SHR (+/−)-alpha-lipoic acid (ALA) 250 µmol/kg/day, SHR + (+/−) ALA 125 µmol/kg/day, SHR + (+) ALA 125 µmol/kg/day, SHR + (−) ALA 125 µmol/kg/day were immunoblotted using the Oxyblot kit; (**b**) The bar graph reports the level of oxidized proteins measured in optical density unit (ODU); (**c**) Sections of kidney processed for the 8-oxo-dG immunohistochemistry (IHC). Arrow: positive nucleus. Calibration bar 25 µm; (**d**) The bar graph shows the quantification of the number of positive cells per high-power field (HPF) (40×). Data are mean ± S.E.M. * *p* < 0.05 vs. WKY C; # *p* < 0.05 vs. SHR C.

To assess the overall level of oxidative modification of proteins in the kidney of rats, we carried out Western blot analyses using the OxyBlot Protein Oxidation Detection kit. A consistent increase in protein oxidation was detected in the renal cortex of SHR versus normotensive WKY rats (Figure 1a,b), suggesting that protein oxidation is related to hypertensive status. The administrations of (+/−)-ALAs and of (−)-ALA 125 µmol/kg/day did not lead to a reduction in oxidation levels of renal proteins compared to hypertensive control (Figure 1a,b). On the contrary, a significant decrease was found in SHR treated with (+)-ALA 125 µmol/kg/day (Figure 1a,b). To determine if there was an increase in oxidative DNA damage, IHC anti-8-oxo-dG was performed in the sections of the kidney (Figure 1c). Figure 1c showed representative images of 8-oxo-dG staining more cells of proximal and distal convoluted tubules than nuclei of the glomerular mesangium. The SHR rats showed an increase of 8-oxo-dG positive nuclei, and intensity of immunoreaction, due to oxidative stress, which also involves nucleic acids (Figure 1c,d). Only the treatment with antioxidant (+)-ALA 125 µmol/kg/day decreased the number of positive cells and the intensity of immunoreaction compared to SHR control rats (Figure 1c,d). Indeed, the administration of (+/−)-ALA 250, (+/−)-ALA 125 µmol/kg/day and (−)-ALA 125 µmol/kg/day did not induce a reduction in the number of 8-oxo-dG positive cells (Figure 1c,d).

**Figure 2:**
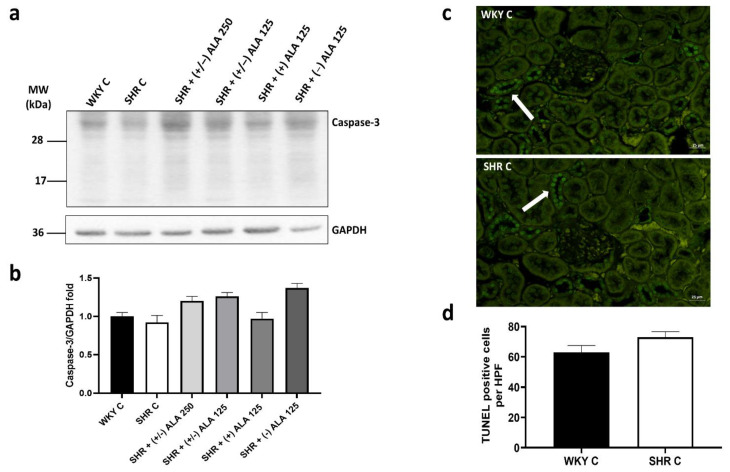
Apoptosis in the kidney. (**a**) Lysates of kidney from WKY C, SHR C, SHR + (+/−) alpha-lipoic acid (ALA) 250 µmol/kg/day, SHR + (+/−) ALA 125 µmol/kg/day, SHR + (+) ALA 125 µmol/kg/day, SHR + (−) ALA 125 µmol/kg/day were immunoblotted using specific anti caspase-3; (**b**) The bar graph indicates the ratio of densitometric analysis of bands and GAPDH levels, used as loading control, considering the WKY C group as reference. Blots are representative of one of three separate experiments; (**c**) Sections of kidney from WKY C and SHR C processed for TUNEL staining. Arrow: apoptotic nucleus. Calibration bar 25 µm; (**d**) Bar graph shows the quantification of the number of positive cells per high-power field (HPF) (40×). Data are mean ± S.E.M.

The presence of oxidative damage in the kidney induced by hypertension did not imply an increase in apoptotic processes. As shown in Figure 2a,b, the expression of caspase-3 (molecular weight 34 kDa) in SHR rats did not change compared to WKY control rats, as well as in the treated groups. Even if an increasing tendency of caspase-3 in animals treated with (+/−)-ALAs and (−)-ALA 125 µmol/kg/day was found compared to the SHR control group, the differences were not statistically significant. Moreover, there was no increase in TUNEL positive cells per field in SHR compared to normotensive WKY rats (Figure 2c,d).

**Figure 3:**
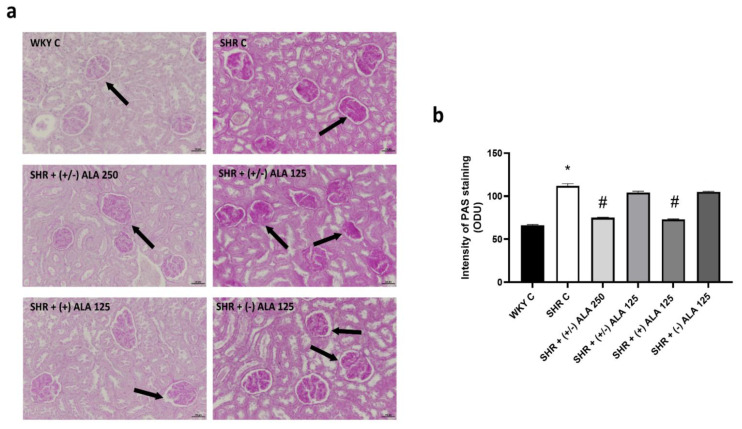
Renal glomerular morphology. (**a**) Renal tissue of WKY C, SHR C, SHR + (+/−) alpha lipoic acid (ALA) 250 µmol/kg/day, SHR + (+/−) ALA 125 µmol/kg/day, SHR + (+) ALA 125 µmol/kg/day, SHR + (−) ALA 125 µmol/kg/day were stained using periodic acid-Schiff (PAS) technique. Arrow: damaged glomerulus. Calibration bar: 50 µm; (**b**) The bar graph shows the quantification of the intensity of PAS staining measured in optical density unit (ODU). Data are mean ± S.E.M. * *p* < 0.05 vs. WKY C; # *p* < 0.05 vs. SHR C.

In hypertensive rats, PAS staining of the kidney showed glomerular structural abnormalities, and several tubules exhibited PAS droplets (Figure 3). As showed by quantification of its staining intensity (Figure 3b), the administrations of (+/−)-ALA 125 µmol/kg/day and (−)-ALA 125 µmol/kg/day in SHR rats did not influence the particularly heavy PAS labeling found in SHR control animals. On the contrary, the compounds (+/−)-ALA 250 µmol/kg/day and (+)-ALA 125 µmol/kg/day were demonstrated to be the most effective. These data were in the direction of the semiquantitative analysis on Masson’s trichrome staining.

**Figure 4:**
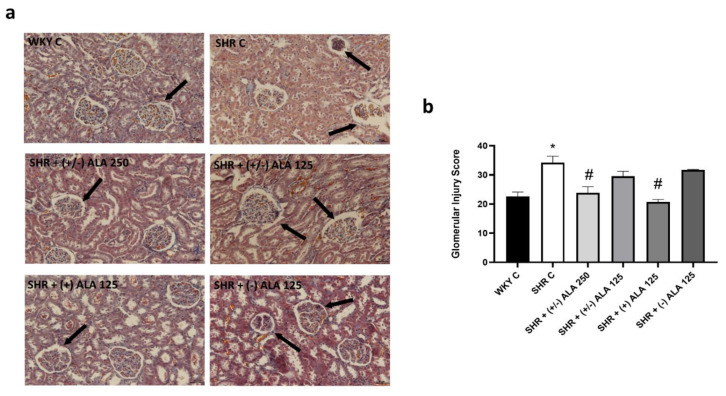
Renal glomerular morphology. (**a**) Renal tissue of WKY C, SHR C, SHR + (+/−) alpha lipoic acid (ALA) 250 µmol/kg/day, SHR + (+/−) ALA 125 µmol/kg/day, SHR + (+) ALA 125 µmol/kg/day, SHR + (−) ALA 125 µmol/kg/day were stained using Masson’s trichrome technique. Arrow: damaged glomerulus. Calibration bar: 50 µm; (**b**) Morphometric analysis to assess the glomerular injury score. Data are mean ± S.E.M. * *p* < 0.05 vs. WKY C; # *p* < 0.05 vs. SHR C.

**Figure 5:**
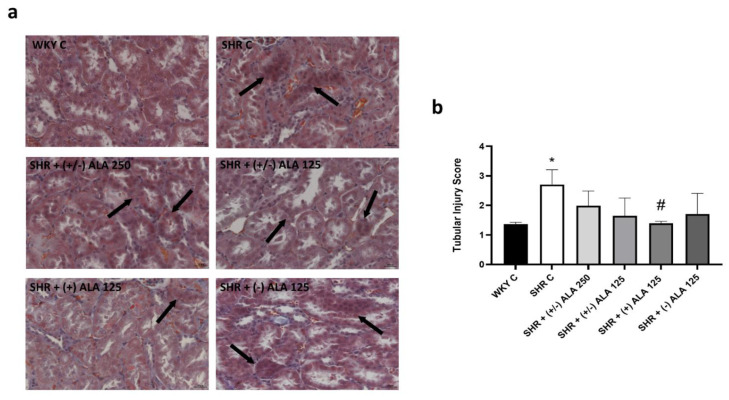
Renal tubules morphology. (**a**) Renal tissue of WKY C, SHR C, SHR + (+/−) alpha lipoic acid (ALA) 250 µmol/kg/day, SHR + (+/−) ALA 125 µmol/kg/day, SHR + (+) ALA 125 µmol/kg/day, SHR + (−) ALA 125 µmol/kg/day were stained using Masson’s trichrome technique. Arrow: damaged tubules. Calibration bar: 25 µm; (**b**) Morphometric analysis to assess the tubular injury score. Data are mean ± S.E.M. * *p* < 0.05 vs. WKY C; # *p* < 0.05 vs. SHR C.

In SHR, the renal morphological and morphometric data showed parenchymal changes consisting of glomerular lesions, characterized by basal wall thickening and atrophy (Figure 4a,b). Besides, tubular alterations were observed both in proximal and distal tubules, characterized by degenerative tubular epithelium, which was thinner and necrotic (Figure 5a). Quantitative analyses of glomerular and tubular damages showed an increase in both GIS and TIS values (Figure 4b and Figure 5b, respectively) in hypertensive rats compared to normotensive ones. The treatments with higher dose of racemic ALA prevented partially the GIS (Figure 4b). Indeed, the most active compound was (+)-ALA 125 µmol/kg/day both at the glomerular and tubular level (Figure 4b and Figure 5b), while (−)-ALA 125 µmol/kg/day was not effective in preventing the damage induced by hypertension (Figure 4b and Figure 5b).

### 3.2. Heart

**Figure 6:**
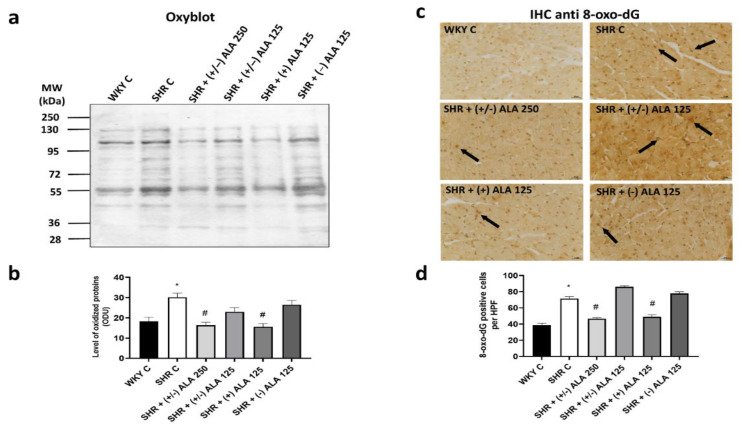
Oxidative stress in heart. (**a**) Lysates of heart from WKY C, SHR C, SHR + (+/−) alpha lipoic acid (ALA) 250 µmol/kg/day, SHR + (+/−) ALA 125 µmol/kg/day, SHR + (+) ALA 125 µmol/kg/day, SHR + (−) ALA 125 µmol/kg/day were immunoblotted using the Oxyblot kit; (**b**) The bar graph reports the level of oxidized proteins measured in optical density unit (ODU); (**c**) Sections of heart processed for the 8-oxo-dG immunohistochemistry (IHC). Arrow: positive nucleus. Calibration bar 25 µm; (**d**) The bar graph shows the quantification of the number of positive cells per high-power field (HPF) (40×). Data are mean ± S.E.M. * *p* < 0.05 vs. WKY C; # *p* < 0.05 vs. SHR C.

The results of Oxyblot kit showed an increase of oxidized proteins in the ventricles of the heart of SHR versus normotensive WKY rats (Figure 6a,b), as well as in kidneys, suggesting that protein oxidation was also related to hypertensive status. Treatment with antioxidants partially decreased the level of oxidized proteins, as shown in rats injected with (+/−)-ALA 250 µmol/kg/day and (+)-ALA 125 µmol/kg/day (Figure 6a,b). In line with the Western blot analysis was the IHC for 8-oxo-dG staining the nuclei of cardiomyocytes (Figure 6c). SHR rats showed an increase both in the number of positive nuclei, and intensity of immunoreaction, due to oxidative stress, which also involved nucleic acids (Figure 6c,d). These nuclei were larger in hypertensive rats than in normotensive ones. The treatment with the antioxidants (+/−)-ALA 250 µmol/kg/day and (+)-ALA 125 µmol/kg/day decreased the number of positive cells and the intensity of the immunoreaction compared to hypertensive control animals, while (+/−)-ALA 125 µmol/kg/day and the (−)-ALA did not induce changes in the number of 8-oxo-dG positive cells per field (Figure 6c,d).

**Figure 7:**
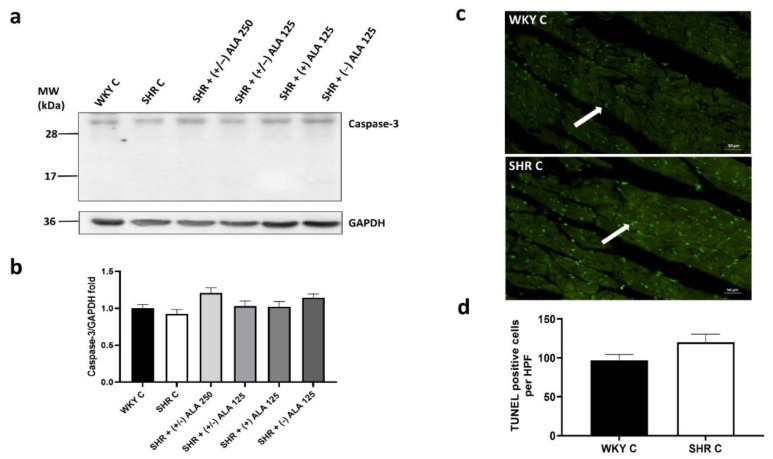
Apoptosis in heart. (**a**) Lysates of heart from WKY C, SHR C, SHR + (+/−) alpha lipoic acid (ALA) 250 µmol/kg/day, SHR + (+/−) ALA 125 µmol/kg/day, SHR + (+) ALA 125 µmol/kg/day, SHR + (−) ALA 125 µmol/kg/day were immunoblotted using specific anti caspase-3; (**b**) The bar graph indicates the ratio of densitometric analysis of bands and GAPDH levels used as loading control, considering the WKY C group as reference. Blots are representative of one of three separate experiments; (**c**) Sections of heart from WKY C and SHR C processed for TUNEL staining. Arrow: apoptotic nucleus. Calibration bar 50 µm; (**d**) The bar graph shows the quantification of the number of positive cells per high-power field (HPF) (20×). Data are mean ± S.E.M.

As well as in the kidney, the apoptosis pathway was not initiated in the heart. As shown in Figure 7a,b the expression of caspase-3 (molecular weight 34 kDa) in SHR rats did not change compared to WKY control rats, as well as in the treated groups. Moreover, there was no increase in TUNEL positive cells in SHR compared to normotensive WKY rats (Figure 7c,d).

**Figure 8:**
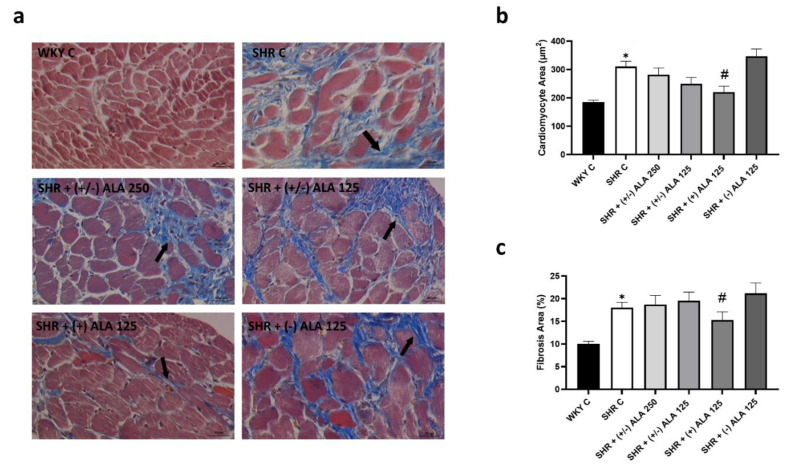
Heart morphology. (**a**) Cardiac tissue of WKY C, SHR C, SHR + (+/−) alpha lipoic acid (ALA) 250 µmol/kg/day, SHR + (+/−) ALA 125 µmol/kg/day, SHR + (+) ALA 125 µmol/kg/day, SHR + (−) ALA 125 µmol/kg/day were stained using Masson’s trichrome technique. Arrow: damaged cardiomyocytes. Calibration bar: 50 µm; (**b**,**c**) Morphometric analysis to evaluate the areas of cardiomyocytes and cardiac fibrosis, respectively. Data are mean ± S.E.M. * *p* < 0.05 vs. WKY C; # *p* < 0.05 vs. SHR C.

Morphometric analyses of the myocardium, focused at the subendocardial level, showed a clear connective tissue accumulated between the cardiomyocytes in SHR rats (Figure 8a). Moreover, an increase of cardiomyocyte areas was reported in SHR rats compared to WKY rats (Figure 8b), as well as hypertrophy of the left ventricular wall, but not the right one. This phenomenon was reduced only by treatment of (+)-ALA 125 µmol/kg/day (Figure 8b), which significantly decreased left ventricular fibrosis (Figure 8c) while the (−)-ALA 125 µmol/kg/day did not have any positive effect either on cardiac hypertrophy nor fibrosis (Figure 8b,c).

## 4. Discussion

Arterial hypertension can be generated by numerous factors, such as lifestyle, physiological and genetic causes [33]. High blood pressure induced mechanical damage along with the vascular system, heart, and kidneys, which are the main organs negatively impaired [33,34]. Indeed, heart failure, cardiac hypertrophy and renal dysfunction are some features of hypertensive end-organ damage developing in SHR rats [34,35].

Our data supported the coexistence of cardiac and renal damage in SHR [20,23,30,36,37,38]. Histologically, we confirmed glomerular and tubular lesions in hypertensive conditions [1,5,23]. Basal wall thickening and atrophy were found at the glomerular level, while the degenerative tubular epithelium was thinner and necrotic. The attempts of reparative mechanisms lead to end-organ damage, principally due to fibrosis [33]. Concerning this, excessive matrix deposition, remodeling, and cardiac hypertrophy become maladaptive responses to prolonged and abnormal hemodynamic stress, due to hypertension [6,7,39]. In accordance, we showed left ventricular hypertrophy and increased fibrosis (histologic hallmarks of cardiac failure) in SHR rats.

Even if caspase-mediated myocardial apoptosis was reported in SHR [40], here we did not have any differences, probably related to duration or severity of hypertension [41].

Moreover, the inflammatory pathway and oxidative stress can be triggered by hypertension. Despite the evidence regarding oxidative stress involvement in hypertension, the mechanisms involved are not well defined [42]. Hypertension is related to the overproduction of ROS, together with decreased NO bioavailability and reduced antioxidant capacity in the brain, vasculature, and kidney [11,12,42,43]. The ROS generated in cardiovascular cells induce pathological vascular injury in blood vessels associated with the accumulation of extracellular matrix protein, endothelial dysfunction, and inflammation, typical features of vascular phenotype in hypertension [8,9,44]. Many experimental models have reported the relationship between oxidative stress and hypertension [45,46,47]. As reviewed by [48] in SHR models, ROS such as superoxide and hydrogen peroxide (H_2_O_2_), as well as oxidation markers such as tyrosine nitration of proteins, production, and excretion of 8-isoprostaglandin F2α (8-iso), MDA, and thiobarbituric acid reactive substances, are increased in vessels, heart, and kidneys. Moreover, renal blood flow and glomerular filtration are reduced in SHR, similarly in obesity-prone and Dahl salt-sensitive rats [48]. Interestingly, hypertensive males have been reported to have greater levels of oxidative stress compared with females [49]. In addition, SHRs exhibit sex differences in blood pressure, with males having higher blood pressure than females [50]; for this reason, we chose males. Previously, an increase of lipid peroxidation and nucleic acid oxidation in plasma, kidney, and heart of SHR rats has been demonstrated [20,24]. Not only the presence of oxidative stress but also elevated endothelial adhesion molecules, such as intercellular adhesion molecule 1 (ICAM-1) and vascular cell adhesion molecule 1 (VCAM-1) expression were found in the heart and the renal endothelium of hypertensive rats [20].

These data highlighted the importance of antioxidant compounds as a support to conventional anti-hypertensive treatments. Clinical randomized studies demonstrated the potential anti-hypertensive effect of antioxidant molecules in the diet both in hypertensive and normotensive subjects [15]. Moreover, prenatal use of natural antioxidants may reverse programming progressions and avoid hypertension of developmental origin [51]. Initially, similar dietary approaches showed a reduction in cardiovascular morbidity and mortality in hypertensive subjects, showing benefits [52,53]; however, later, large clinical trials, including the SU.VI.MAX study showed no improvement in blood pressure with antioxidant therapy [54].

Generally used antioxidants include Vitamins A, C, and E, L-arginine, flavonoids, and mitochondria-targeted agents (Coenzyme Q10, acetyl-L-carnitine, and ALA) [15]. ALA is a dithiol compound produced from octanoic acid in mitochondria. The in vitro and in vivo properties of ALA have been widely revised [26,55,56,57], in particular its antioxidant potential, acting as metal chelators, free radical scavengers, repairer of oxidized injury and regenerator of antioxidants defense, such as glutathione (GSH) and catalase activity, vitamins C and E [26,58,59]. The contribution in metabolic pathways related to mitochondria, in cell signaling that may improve coupling of endothelial nitric oxide synthase (eNOS), and in anti-inflammatory actions are other relevant benefits found with ALA supplementation [55,60]. For such reasons, this compound has received great consideration as an antioxidant in the management of diabetic problems such as neuropathy, retinopathy, and other vascular diseases [61]. ALA was also effective in preventing acute kidney injury [62], glomerular injury caused by diabetes mellitus [63], and the toxic effects of acetaminophen [64], cisplatin [65], and methotrexate [66] on the kidney in rats. In addition, in unilateral ureteral obstruction mice treated with ALA, only moderate, histologically identifiable renal injury and minimal fibrosis were reported [67]. At the molecular level, the expression of extracellular matrix (ECM) proteins, fibrogenic and inflammatory factors (transforming growth factor-β1 and monocyte chemoattractant protein-1), and epithelial-to-mesenchymal transition markers (E-cadherin and alpha-smooth muscle actin) was much lower in ALA-treated than in non-treated mice [67]. Regarding results in SHR rats, renal histopathological examination showed that ALA supplementation ameliorated glomerular damage and prevented vascular damage [68]. Moreover, ALA-treated animals showed a significant reduction in urine protein levels, N-acetyl-β-(D)-glucosaminidase activity, and significant increase of creatinine clearance, particularly in SHR at 28 weeks [68]. In patients with autosomal dominant polycystic kidney disease, ALA was suggested as an anti-inflammatory and antioxidant nutraceutical compound with few side effects to reduce the related cardiovascular risk factors [69]. Moreover, the long-term intermittent treatment with ALA prevented body weight gain and reduced metabolic and cardiac alterations (i.e., atherosclerosis), corroborating its protective properties on the cardiovascular system [70,71]. Besides, studies in diabetic rats and other different hypertensive animal models revealed the potential for ALA supplementation to reduce blood pressure [72,73,74,75] and to improve baroreflex sensitivity in rats with renovascular hypertension [76]. In line with our previous published data [20], Vasdev et al. [72] showed no effect of ALA (at 500 mg/kg feed for nine weeks) on body weight during supplementation in SHR rats. Contrary to our results, the ALA supplementation did attenuate hypertension measured by tail-cuff methodology [72], as was similarly reported by Midaoui and Champlain [77] in rats fed with the glucose solution and ALA (500 mg/kg feed). Concerning the mechanisms, they speculated that ALA increased the free sulfhydryl groups of calcium channels, inducing a decrease in cytosolic free calcium, vascular tone, and hypertension [72]. In addition, the authors postulated that the antihypertensive effects of ALA are associated with an attenuation of oxidative stress in the aortic vessel and the preservation of glutathione peroxidase activity in the plasma of glucose-treated rats [77].

Moreover, ALA was recognized to have antiviral effects by reducing not only oxidative stress but also the nuclear factor kappa-light-chain-enhancer of activated B cells (NF-kB) activation [78]. Besides, ALA increased the human host defense activating ATP-dependent K+ channels, and thus the increased K+ in the cell raised the intracellular pH. As a consequence, the entry of the virus into the cell decreased. For these reasons, ALA can increase human host defense against SARS-CoV-2 by increasing intracellular pH and reducing oxidative stress [78].

Even if this compound demonstrated a powerful antioxidant activity in vitro, a short half-life and a modest oral bioavailability were found in vivo [79]. Indeed, therapeutic efficacy is relatively low due to its pharmacokinetic limitation: hepatic degradation, reduced solubility, and gastric instability. However, ALA’s liquid preparations and new amphiphilic matrices’ formulations have significantly enhanced ALA bioavailability and, consequently, therapeutic efficacy [26]. Better pharmacokinetic parameters were found in the R-enantiomer of ALA compared to S-ALA [80]. According to other studies [25,81,82], we found greater effectiveness of (+)-ALA compared to (+/−)-ALA in reducing oxidative stress, demonstrated by the reduction of oxidized proteins levels, and by 8-oxo-dG expression, as well as cardiac and renal damages induced by hypertension. However, the limitation of our study is that we did not assess the effects of various forms of ALA on SHR renal and cardiac functions yet. Thus, further studies are necessary. The advantage in using (+)-ALA, as compared to the racemic form, may be linked to an amplified bioavailability and biological activity of this enantiomer that enhanced the antioxidant activity in SHR animals. The supplementation of lipoic acid as natural antioxidant has already been demonstrated to be safe in clinical trials [83], and to have multiple beneficial effects [57]. In particular, (+)-ALA showed the most pronounced activity, with extremely few acute and subchronic toxicities, compared to all the forms of ALA [84].

## 5. Conclusions

Gathering up data highlights the greater effectiveness of (+)-ALA compared to racemic form in reducing oxidative stress, cardiac and renal damages in SHR. To conclude, these findings propose (+)-ALA as one of the more appropriate antioxidant molecules for slowing down organ alterations associated with hypertension.

## Data Availability

Not applicable.
